# Diagnostic and Therapeutic Strategy in Anaplastic (Malignant) Meningioma, CNS WHO Grade 3

**DOI:** 10.3390/cancers14194689

**Published:** 2022-09-26

**Authors:** Vincenzo Di Nunno, Caterina Giannini, Sofia Asioli, Alfredo Conti, Julia Furtner, Damiano Balestrini, Alicia Tosoni

**Affiliations:** 1 Department of Oncology, AUSL Bologna, 40139 Bologna, Italy; 2 Department of Laboratory Medicine and Pathology, Mayo Clinic, Rochester, MN 59005, USA; 3 Department of Biomedical and Neuromotor Sciences (DIBINEM), University of Bologna, 40139 Bologna, Italy; 4 Pituitary Unit, IRCCS Istituto delle Scienze Neurologiche di Bologna, 49139 Bologna, Italy; 5 Dipartimento di Scienze Biomediche e Neuromotorie (DIBINEM), IRCCS Istituto delle Scienze Neurologiche di Bologna, University of Bologna, 40139 Bologna, Italy; 6 Department of Biomedical Imaging and Image-Guided Therapy, Medical University of Vienna, 1090 Vienna, Austria; 7 Radiotherapy Department, AUSL-IRCCS Scienze Neurologiche, 40139 Bologna, Italy; 8 UOC Oncologia Sistema Nervoso, IRCCS Istituto delle Scienze Neurologiche di Bologna, 40139 Bologna, Italy

**Keywords:** meningioma, anaplastic meningioma, NF2

## Abstract

**Simple Summary:**

Only 1% of all meningioma diagnosis is classified as malignant (anaplastic) meningioma. Due to their rarity, clinical management of these tumors presents several gaps. In this review, we investigate current knowledge of anaplastic meningioma focusing on their pathological and radiological diagnosis, molecular assessment, and loco-regional and systemic management. Despite the current marginal role of systemic therapy, it is possible that the increasing knowledge of molecular altered pathways of the disease will lead to the development of novel effective systemic treatments.

**Abstract:**

*Background*: Meningiomas are the most common primary central nervous system malignancies accounting for 36% of all intracranial tumors. However, only 1% of meningioma is classified as malignant (anaplastic) meningioma. Due to their rarity, clinical management of these tumors presents several gaps. *Methods*: We carried out a narrative review aimed to investigate current knowledge of anaplastic meningioma focusing on their pathological and radiological diagnosis, molecular assessment, and loco-regional and systemic management. *Results*: The most frequent genetic alteration occurring in meningioma is the inactivation in the neurofibromatosis 2 genes (merlin). The accumulation of copy number losses, including 1p, 6p/q, 10q, 14q, and 18p/q, and less frequently 2p/q, 3p, 4p/q, 7p, 8p/q, and 9p, compatible with instability, is restricted to NF2 mutated meningioma. Surgery and different RT approaches represent the milestone of grade 3 meningioma management, while there is a marginal role of systemic therapy. *Conclusions*: Anaplastic meningiomas are rare tumors, and diagnosis should be suspected and confirmed by trained radiologists and pathologists. Despite the current marginal role of systemic therapy, it is possible that the increasing knowledge of molecular altered pathways of the disease will lead to the development of novel effective systemic treatments.

## 1. Introduction

Meningiomas are primary central nervous system (CNS) tumors originating from the arachnoid cells located on the inner surface of the dura [1]. These are the most common primary CNS malignancies accounting for 36% of all intracranial tumors [2] and are in most cases benign (CNS WHO grade 1). By contrast, anaplastic (malignant) meningiomas (malignant meningiomas: MMs) are rare tumors, representing 1% of all meningiomas [3]. Different from low-grade meningiomas that occur most often in women and are associated with a relatively good outcome, MMs are more frequent in men and have a poor prognosis with reported five-year survival rates of 28–61% [4].

Patients with a grade 3 meningioma can be divided into two groups: patients with primary MM who receive a diagnosis of MM at their first surgery and patients with secondary MM in whom the MM is the result of transformation from a lower grade tumor [5,6]. The prognosis of patients with primary MM has been shown to be favorable compared to those with secondary MM in multiple retrospective series [5,7,8,9].

In a recent published series of 51 patients with primary and secondary MM, the time to grade 3 transformation from previous diagnosis of grade 1 or 2 meningiomas was 5.5 years (range 0.5–22 years) [10]. In these same series it emerged that patients with primary or secondary MM did not differ significantly in overall survival and risk of progression [10].

Given the rarity of this tumor, only few clinical prognostic factors have been identified, including: homogeneous contrast enhancement on magnetic resonance imaging [11], a gross total resection at surgery [6,8,11,12,13,14], and radiotherapy (RT) adjuvant treatment [3,14,15].

## 2. Search Strategy

One of the most important limits of the present article is related to the research of original data focusing exclusively on anaplastic meningioma. Indeed, the majority of data are derived from prospective and retrospective series assessing all-grade meningiomas or, in the case of studies oriented on treatment approaches, recurrent meningiomas. We tried to summarize available evidence on MM adopting a systemic and internal revision of articles included in the text.

Even if this is not a systematic review, we adopted a search protocol to optimize the research of studies including patients with grade 3 meningiomas. We searched English-written articles published on PubMed/Medline, Cochrane Library, and Scopus until the 1 May 2022. The keywords adopted for the research were: “anaplastic meningioma” OR “grade 3 meningioma” OR “grade III meningioma” OR “malignant meningioma”. We were interested only to the following article subtypes: “*Clinical Study, Clinical Trial, Clinical Trial Protocol, Clinical Trial, Phase I, Clinical Trial, Phase II, Clinical Trial, Phase III, Clinical Trial, Phase IV, Guideline, Meta-Analysis, Multicenter Study, Observational Study, Randomized Controlled Trial, Validation Study*”.

By this approach, we were able to select 9686 possibly relevant articles. After an initial revision carried out by all the authors, the following articles were selected for each sections:

- Clinical symptoms: 15 articles selected [8,9,12,14,16,17,18,19,20,21,22,23,24,25,26];
- Pathology: 20 articles selected [1,27,28,29,30,31,32,33,34,35,36,37,38,39,40,41,42,43,44,45];
- Radiological features: 12 articles selected [46,47,48,49,50,51,52,53,54,55,56,57];
- Surgery: 17 article selected [10,58,59,60,61,62,63,64,65,66,67,68,69,70,71,72,73];
- Radiation therapy: 20 articles selected [74,75,76,77,78,79,80,81,82,83,84,85,86,87,88,89,90,91,92,93];
- Systemic treatment: 22 articles selected [91,93,94,95,96,97,98,99,100,101,102,103,104,105,106,107,108,109,110,111,112,113,114].

## 3. Clinical Symptoms

Symptoms and clinical presentation of MM are strongly correlated with the localization of primary tumors [19,20]. These tumors can manifest with symptoms related to mass effect, increased intracranial pressure, and focal symptoms related to compression of cranial nerves [19,20]. Anaplastic (malignant) meningiomas frequently occur in the convex surface of the cerebellum, parietal region, and rarely also in the spinal cord [12,16,17,18,25]. These tumors are extremely rare in children and are diagnosed mainly in adult patients; however, the survival is shorter in children compared to adults [22]. Anaplastic (malignant) meningiomas can be extremely invasive and destroy the surrounding bone and extracranial soft tissues [23,25,26]. The recurrence rate of these tumors is higher than 90%. Distant metastases are possible but rare and have been described in about 3% of cases [8,14,24]. The lung is a frequent site of distant spread; however, liver and lymph nodes metastases have also been reported [8,14,21,24].

## 4. Pathology

Meningiomas are the most common intracranial/extraparenchymal tumors [32] and include a large spectrum of tumors with varying histopathological features ranging from benign (CNS World Health Organization/WHO grade 1) tumors to atypical (CNS WHO grade 2) and anaplastic (malignant) tumors (CNS WHO grade 3). Anaplastic (malignant) meningiomas, CNS WHO grade 3, are the least common, accounting for 1–3% of meningiomas (WHO CNS 2021) and include three different subtypes: anaplastic (malignant), rhabdoid, and papillary (WHO CNS 2021). Similar to other tumors in the 2021 WHO CNS tumors classification, they can be diagnosed either based on histopathological findings or a combination of morphological and molecular findings.

### 4.1. Histopathological Diagnosis

Anaplastic (malignant) meningiomas are defined as meningiomas, which show (1) markedly elevated mitotic activity (20 or more mitoses in 10 consecutive high-power fields each of 0.16 mm^2^, at least 12.5 per 1 mm^2^); (2) frank malignant cytology, resembling carcinoma, melanoma, or sarcoma; (3) harbor *TERT* (telomerase reverse transcriptase [1]) promoter mutation; and (4) harbor *CDKN2A* (cyclin-dependent kinase inhibitor [1]) and/or *CDKN2B* homozygous deletion [1]. Extensive necrosis is frequently observed in aplastic (malignant) meningioma, as is parenchymal brain invasion. Malignant features can be present either at first resection or at recurrence.

The diagnosis of meningioma can be confirmed by immunohistochemical stains since most meningiomas express *EMA* (epithelial membrane antigen, [33]), progesterone receptor, and vimentin. Both EMA and progesterone receptor stains, however, can be faint, focal, or absent, particularly in high-grade subtypes [33]. A stain which can be quite helpful is somatostatin receptor 2A (*SSTR2A,* [33]), which shows strong and diffuse positivity in most meningiomas and whose expression is typically retained in grade 3 examples. This stain, however, needs to be evaluated carefully in the context of the overall histopathological, immunohistochemical, and molecular findings since *SSTR2A* can be expressed in a variety of other tumors occurring in the meninges, including solitary fibrous tumor.

Assessment of proliferative activity can be facilitated by the Ki67 stain, which highlights the most proliferative foci and therefore facilitates mitotic count. One caveat is, however, the presence of macrophages and tumor-infiltrating lymphocytes (TIL), which can spuriously increase the Ki67 counts [27]. Compared to mitotic counts, the assessment of frank anaplasia as a criterion for the diagnosis of anaplastic (malignant) meningioma is subject to greater interobserver variability and lower reproducibility. Extreme anaplasia and true sarcomatous (metaplastic) differentiation may make the diagnosis extremely challenging [33]. In both cases, molecular studies (see below) can help to identify a molecular signature supportive of the diagnosis.

Loss of *H3* (histone 3, [29,31,37]) p.K28me3 (K27me3) has been reported in 10–20% of anaplastic (malignant) meningiomas, and it could be associated with decreased overall survival [29,31,37]. However, a recently published study failed to show a significant OS difference between patients with retained or lost H3-K27me3 [115].

### 4.2. Rhabdoid and Papillary Meningiomas

While in previous WHO classification rhabdoid and papillary meningiomas were considered malignant (WHO grade 3) simply based on their histological features, for both tumors now the presence of additional features, which fulfil criteria for classification as anaplastic (malignant) meningioma, irrespective of the rhabdoid or papillary phenotype, is required for a CNS WHO grade 3 designation.

Rhabdoid meningioma shows the presence of rhabdoid cells, which are plump cells with eccentric nuclei, open chromatin, macronucleoli, and prominent eosinophilic paranuclear inclusions [38]. Most of them are highly proliferative and usually fulfil the criteria for anaplastic meningioma grade 3 according to CNS WHO 2021.

Vaubel RA et al., showed that some meningiomas may show rhabdoid features only focally or lack high mitotic activity, and the behavior of these tumors is more in line with their histologic grade than with the rhabdoid appearance; they should therefore be graded similarly to non-rhabdoid meningiomas. The presence of rhabdoid features should, however, still be reported as some of these tumors may still behave aggressively. Close follow-up of these patients is required [44].

Papillary meningioma is characterized by a predominant perivascular papillary/pseudopapillary pattern [1,34]. The tumor cells typically are arranged in a perivascular pseudorosette-like pattern and at times show rhabdoid morphology. The presence of focal papillary architecture and/or the absence of other high-grade features in a meningioma with papillary architecture is not sufficient for designating the tumors as CNS WHO grade 3 [1]. Rhabdoid and papillary meningiomas can occur both in children and adult patients.

*BAP1* (BRCA1-associated protein, [41]) mutations resulting in loss of *BAP1* and loss of nuclear expression have been reported both in rhabdoid and papillary meningioma and can be associated with *BAP1* tumor predisposition syndrome. In the study by Shankar et al., patients whose tumors were *BAP1* negative had reduced time to recurence and required intensive clinical management [41].

### 4.3. Anaplastic (Malignant) Meningioma

The most frequent genetic alteration occurring in meningioma is the inactivation in the neurofibromatosis 2 genes (merlin) on chromosome 22q, which occurs in approximately 50% of meningiomas.

Mutations occurring in the non-*NF2* (neurofibromatosis type 2 gene, [45])-mutated meningioma include *TRAF7* (Tumor Necrosis Factor Receptor Associated Factor 7, [45]), *AKT* (protein kinase B), *SMO* (smoothened frizzled class receptor, [45]), and *PIK3CA* (phosphatidylinositol-4,5-bisphosphate 3-kinase catalytic subunit alpha, [45]) genes, which are strongly related to the meningioma subtypes and are typically associated with low-grade (CNS WHO grade 1) meningiomas [45]. This paper concentrates its attention on *NF2* mutated meningioma, whose spectrum spans from CNS WHO grade 1 to grade 3 tumors.

Allelic losses in 22q12.2 regions, encoding the *NF2* gene, are the most common abnormalities in this group of tumors (40–60% of cases). 22q loss of heterozygosity incidence increases with meningioma grade (75–85% in grade 3 meningioma) [39,43]. A double-hit mechanism is involved in the inactivation of merlin (69-kDa moesin–ezrin–radixin-like protein encoded by *NF2* gene): 22q loss of heterozygosity followed by a second hit on the remaining gene (nonsense or frameshift or missense mutations or affecting splice sites, or interstitial deletions).

According to the CNS WHO classification, more than 30% of *NF2*-mutated meningiomas are grade 2–3 and recur more frequently than the others. *NF2*-mutated meningiomas show high chromosome instability during progression.

The accumulation of copy number losses, including 1p, 6p/q, 10q, 14q, and 18p/q and, less frequently, 2p/q, 3p, 4p/q, 7p, 8p/q, and 9p, compatible with instability is restricted to *NF2*-mutated meningioma. Recurrent genomic alterations, mainly involving *CDKN2A/CDKN2B* locus loss on 9p, are found frequently in meningiomas at recurrence as well as at progression and are associated with prognosis. *CDKN2A* and/or *CDKN2B* homozygous deletion is now considered sufficient for a CNS WHO grade 3 designation [28,42].

Gains of chromosomal arms on 1q/9q/12q/15q/17q/20q are less common and mostly found in specific low-grade subtype meningiomas [1].

*TERT* promoter mutations also occur mostly (although not exclusively) in *NF2*-altered meningioma and, although uncommon, are highly associated with grade and decreased time to recurrence/progression [40]. In particular, hotspot mutations (C228T and C250T) in the *TERT* promoter were detected in 20% of WHO grade 3 meningiomas compared to 1.7% and 5.7% of grade 1 and 2 meningiomas, respectively [1,40], and in 6.4% in a large cohort of meningiomas [30].

Maier et al. [36] reported that *TERT* promoter mutations can occur independently of malignant progression in meningioma. *TERT* promoter mutation was most often present from the primitive tumor tissue across recurrences in a consecutive single-center cohort of malignant meningioma.

Assessment of *TERT* promoter status has now been added as a criterion for a diagnosis of CNS WHO grade 3 meningioma independent of the histopathological findings. 

While this brief discussion is limited to tumors which are diagnosed as anaplastic (malignant) meningioma in 2021 WHO CNS tumor classification, as it pertains to the full spectrum of meningioma, a critical clinical need is how to distinguish those patients with low or no risk of recurrence from those with an intermediate risk among tumors at present in the spectrum of CNS WHO grade 1 and 2 meningiomas. Integrated morphological and mostly molecularly based meningioma classifications incorporating copy number mutational profile and whole-genome methylation profile are being developed to better predict patient outcomes and inform clinical decision-making [30,35,36].

## 5. Radiological Features

On computed tomography (CT), meningiomas present usually as slightly hyperdense, extra-axial, well-circumscribed, dura-based masses that may show intratumoral calcifications, especially in slow-growing subtypes, frequently discovered incidentally. Adjacent remodeling of the skull or hyperostosis can be found [46,56].

To further characterize the tumoral lesion, gadolinium-enhanced magnetic resonance imaging (MRI) is the method of choice for the diagnosis and response assessment in meningioma patients [19,50].

Herein, meningiomas in general are hypo- to isointense on T1-weighted images and hypo- to hyperintense on T2-weighted images showing homogenous vivid contrast enhancement after intravenous application of contrast enhancement. Central necrosis, cysts, or perifocal oedema are not indicative to determine the tumor grade as they can occur in both benign and malignant meningiomas. In meningiomas, the adjacent dura is often thickened, and contrast-enhancing is known as dural tail, and a CSF (cerebrospinal fluid) cleft is often seen between the extra-axial tumoral mass and the adjacent brain cortex, which, however, can also be seen in other extra-axial masses such as dural metastases or solitary fibrous tumors [50].

**Figure 1:**
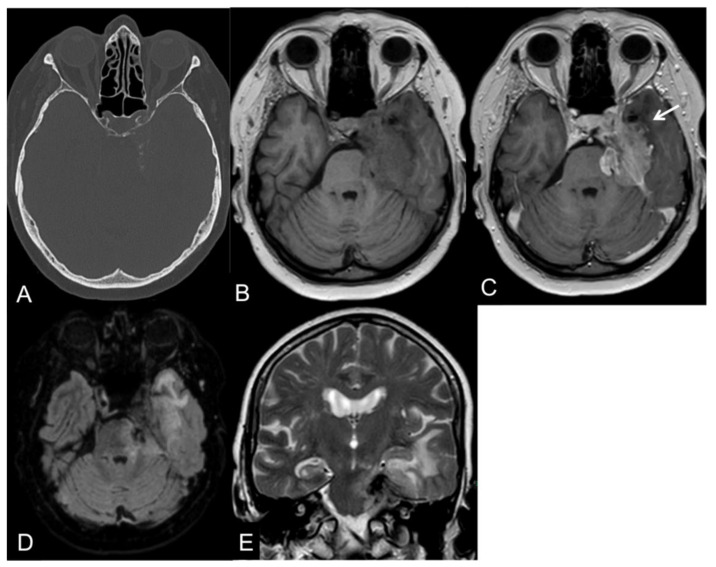
CT and MR images of a 69-year-old female patient with an anaplastic petroclival meningioma (III). The tumor shows calcifications in the CT scan (**A**). On T1-weight images, the intratumoral signal is iso- to hypointense in comparison to the gray matter (**B**) with a vivid contrast enhancement after intravenous gadolinium-based contrast enhancement (**C**) that extends into the adjacent brain parenchyma, representing parenchymal infiltration (arrow). On fluid-attenuated inversion recovery (FLAIR; (**D**)) and T2-weighted images (**E**), the tumor presents a central low signal intensity representing the intratumoral calcifications, an absence of the CSF cleft, and a moderate perifocal edema.

Anaplastic (malignant) meningiomas are characterized by aggressive behavior presenting as loss of the CSF cleft, the missing demarcation between the tumoral mass and the adjacent brain parenchyma, and invasion of the surrounding tissues [53]. An example of a CNS WHO grade 3 meningioma is illustrated in Figure 1.

Diffusion-weighted imaging (DWI) is reported to aid in the differentiation between benign and malign meningiomas representing decreased apparent diffusion coefficient (ADC) values in the high-grade subtypes; however, the results are controversial [49,52,55].

While intratumoral relative cerebral blood volume (rCBV) does not differentiate between benign and malignant meningiomas, MR perfusion is increased in the perifocal oedema in malignant meningiomas due to the local infiltration of tumor cells [57].

In MR-spectroscopy, meningiomas are characterized by increased choline and alanine peaks, whereas N-acetyl aspartate and creatine peaks are reduced [46].

Recently, radiomics is increasingly gaining importance in neuro-oncological imaging correlating quantitative radiological features with, e.g., histopathological or molecular tumor subtypes. Several studies have reported a potential role of radiographic features, e.g., shape or texture in predicting tumor grade in noninvasive meningioma (WHO grade I meningioma) [47,51,54].

The Response Assessment in Neuro-Oncology (RANO) Meningioma Working Group proposed response criteria, especially for clinical trials in meningioma patients based on the standardized Brain Tumor Imaging Protocol including a 3D T1-weighted contrast-enhanced MR sequence with a slice thickness of ≤1.5 mm [48,50]. These response criteria are solely eligible for fast-growing meningiomas (showing a 15% increase in the sum of the products of perpendicular diameters within the last 6 months) or if a new lesion has developed. For slow-growing meningiomas, more sensitive indicators of response, such as a change in the rate of growth, may be more appropriate [50].

Complete response (CR) is defined as an absence of all contrast-enhancing lesions for at least 8 weeks. Given the low rate of response expected, particularly in grade I meningiomas, the category minor response (MR) is determined as a reduction in the product of the maximum perpendicular diameters of 25% or more but less than 50% has been added. If the decrease exceeds 50%, it is characterized as partial response (PR). In either case, the reduction has to sustain for at least 8 weeks. An increase by ≥25% in the sum of the product of perpendicular diameters of target lesions compared with the smallest tumor measurement, any new lesion, or considerable progression of nontarget lesions are specified as progressive disease (PD). If none of the above-mentioned criteria is suitable, it is defined as a stable disease (SD). Besides the radiological criteria, the usage of corticosteroids and the clinical status has to be taken into account [50].

## 6. Surgical Approach

Although incidentally found, asymptomatic meningiomas managed by observation typically present a rapid growth that requires a shift in the management toward surgical resection to relieve mass effect. Thus, surgical treatment is generally considered the chief treatment for grade 3 meningiomas, whereas preresection biopsy is not generally indicated for meningiomas of any grade. Indeed, surgical resection relieves mass effect and allows histopathological characterization of the tumor. 

As a rule, complete resection of the tumor, together with the dural attachment, should be pursued with surgery. Indeed, for meningiomas, the extent of resection (EOR) is strongly associated with the probability of recurrence, and it is generally graded according to the Simpson scale [70]. According to the Simpson grading system, EOR is categorized into five classes (Table 1), where Grades I–III are classified as gross total resection (GTR), and Simpson Grades IV–V constitute subtotal resection (STR) [58,61,69]. Recently, a sixth category, Grade 0, has been proposed in which there is complete tumor removal plus an additional 2–3 cm from tumor insertion site with good results [67].

In several studies, the extent of resection is an independent prognostic factor for progression-free survival and local control [59,63,70]. Thus, the goal of surgery for meningiomas is GTR.

However, local control in MM remains dismal even after GTR, with 5-year recurrence rates as high as 72–94% [59,63,68], making the role of GTR in these tumors unclear.

Recently, Orton et al. [14] retrieved 755 adult patients with MM identified from the National Cancer Database (NCDB), a hospital-based cancer registry including data of approximately 70% of cancer diagnoses in the United States [66]. In this study, postoperative RT turned out to be associated with improved survival (log-rank *p* < 0.01), whereas a GTR showed only a trend toward improved survival, not meeting statistical significance (3-year survival was 63.1% for those undergoing GTR vs. 53.4% for those undergoing STR, log-rank *p* = 0.06). Noteworthy, the best outcome was achieved in patients receiving GTR plus RT, while those undergoing neither GTR nor RT fared the worst (log-rank *p* < 0.01). Moreover, for patients receiving a STR, survival was improved with the addition of RT (log-rank *p* < 0.01). Importantly, survival of patients undergoing STR and receiving RT was similar to that of those who had a GTR and did not reknot followed by RT (log-rank *p* = 0.28) [14].

Similarly, the impact of EOR in MM has been questioned by Sughrue et al. [71], who suggested that patients may achieve better results after subtotal resection followed by adjuvant RT [71]. Indeed, if it is reasonable pursuing a GTR in such aggressive tumors, it may not be achievable in all patients without risk of significant morbidity. Over-aggressive surgery can sometimes cause major complications with consequent negative effects on quality of life. For instance, the frequent location of malignant meningiomas in the parasagittal area prevents a grade I–II resection for the impossibility to resect a patent sagittal sinus or because of the large extension of the lesion deep inside the interhemispheric scissure along the falx. Similarly, GTR of skull base meningiomas can be difficult because of tight relationships with critical neurovascular structures.

Accordingly, because of the rarity of this disease and the extremely limited evidence available in the literature, the role of EOR for MM has not been adequately investigated so well as the impact on postoperative performance status, and this fact should be taken into consideration by the surgeon when formulating the surgical strategy for individual patient [59,60]. Generally, maximal safe resection followed by RT could be considered the best treatment option for MM, but not at the price of major morbidities.

It has been reported that around 50% of MM recur 2–3 years after initial resection independently of EOR [8,12,71,73]. Surgery remains the chief treatment modality for recurrent tumors. Sughrue et al., have shown an overall survival benefit of salvage surgery after the first recurrence, with a median OS (overall survival) of 53 months with vs. 25 months without (*p* = 0.02) [71]. The time between the first and second surgery in the series of Champeux et al., was 1.3 years [12]. On average, patients with MM undergo three surgical operations [8,71]. However, there is no evidence that more than two operations have still a beneficial profile over the risk of complications, including infection and wound closure problems due to multiple surgeries and irradiations.

In a recently published series, the 30-day mortality rate of 51 patients with primary and secondary malignant meningioma was 11.8% [10]. Furthermore, in this same series, surgery was followed by a modified Rankin Scale score only in a limited percentage of patients [10].

Surgery-related complications in MM are likely common but have been reported in detail only by Sughrue et al. [71]. They found 41% (26/63) of patients acquired medical (10%) or surgical (31%) complications, 60% of which were considered major, such as decreased level of consciousness, cranial nerve deficits, and motor and language deficits. One main risk in meningioma surgery is venous thromboembolism. In a large study on the topic collecting 581 patients, 20% of whom affected by atypical or anaplastic meningioma, a 7% risk of venous thromboembolic events was found [64]. The risk was not associated with the histopathology of tumors.

The preventive use of anticonvulsants in meningioma surgery is not recommended. In a meta-analysis comprising 19 studies and 698 subjects with meningiomas (most of which were benign), routine use of anticonvulsants in meningioma did not prove beneficial for the prevention of both early and late postoperative seizures [65]. However, epilepsy in MM is common, and patients who present with seizures preoperatively or develop seizures during the follow-up period required long-term antiepileptic treatment [62,72].

In conclusion, the evidence on the best surgical management and postoperative care is limited; further collaborative studies are strongly encouraged.

## 7. Radiotherapy

External RT after surgery is considered a standard of care in patients with CNS WHO grade 3 meningioma [88,91] in the case of STR as well as in the case of GTR. Furthermore, RT can be considered in patients unsuitable for surgery [91]. The optimal RT planning is individualized and depends on meningioma size, proximity to critical structures, and prior radiation treatment received [87,88].

The role of RT adjuvant to surgery in CNS WHO grade 3 meningiomas has been recently investigated in a meta-analysis [93]. The authors identified 21 studies investigating RT after surgery. Notably, 14 of 21 studies compared radiation therapy to observation following resection. RT after surgery seemed to prolong the survival of patients with grade 3 meningioma by about 2 years (60 months versus 36 months). Notably, the majority of studies included in the analysis reported a better survival trend toward the administration of RT after surgery; however, some studies failed to identify a significant difference in terms of overall survival [93]. It is important to observe that the quality of evidence of each study resulted in “very low quality” adoption of the GRADE system (Grading of Recommendations, Assessment, Development, and Evaluation) [93]. In addition, there was a significant risk of bias because most of the trials included patients with both grade 3 and grade 2 meningiomas. Moreover, in the retrospective trial, RT was not offered to patients with negative prognostic factors such as bad postoperative performance status, favoring better results in the subpopulation treated with adjuvant radiation therapy.

Two prospective clinical trials are investigating the role of adjuvant RT in high-risk meningioma [75,77]. The RTOG (Radiation Therapy Oncology Group) 0539 was a phase II study exploring the clinical outcome of patients receiving intensity-modulated radiation therapy (IMRT) following surgery with any resection extent [77]. High-risk meningioma was defined as grade 3 meningiomas, recurrent grade 2 meningioma, or newly diagnosed grade 2 meningioma recurrent after subtotal resection. All patients received 60 Gy in 30 fractions. In this population, the 3-year progression-free survival was 58.8%; however, a longer follow-up is required to provide definitive results [77].

The EORTC (European Organization for Research and Treatment of Cancer) 22042 is a phase II observational study investigating the role of adjuvant radiation therapy on high-risk meningioma [75]. No data about the cohort of patients with grade 3 meningioma are still available [75].

Few studies investigated the role of adjuvant stereotactic radiotherapy (SRS) in patients with grade 3 meningiomas [74,78,79,82,89,93]. Overall, these were small, nonrandomized studies investigating SRS in patients with resected (any resection extent) meningiomas without a distinction between grade 2 and grade 3 subtypes [74,78,79,82,89,93]. Among these studies, the majority failed to show a significant improvement in terms of progression-free survival (PFS) or OS. Only one study [89] demonstrated an impressive long-term PFS with SRS; however, this same study presented a very large proportion of small residual malignant tumors as compared to other similar trials [89].

In conclusion, adjuvant RT in grade 3 meningiomas is considered a standard of care even if the quality of studies supporting this statement is low and weighted by the inclusion of patients with different pathological and clinical features; in addition to not using the most updated WHO definition, often both atypical and MM were included. Early trials demonstrate that higher RT doses appear to improve local tumor control [80,86]. Recently, the most frequently used doses starting from 54 to 60 Gy in 1.8–2.0 Gy fractions. It is to be underlined that the most recent study protocols adopt a scheme of 60 Gy in 30 fractions, such as RTOG 0539 trial (NCT00895622) as well as EORTC 22042-26042 trial (NCT00626730), which also adds a 10 Gy boost in case of STR. Using doses above 60 Gy demonstrated a better outcome in one study using combined photons and protons [85] and could be a reasonable approach to obtain better control of the residual disease.

The CTV (clinical target volume) is identified as all the surgical bed with a margin including the adjacent dura. It usually included 1 to 2 cm of dura around the cavity, while RTOG 0539 used a higher dose with a CTV with a 1 cm margin and a lower dose with a 2 cm margin.

There are no studies investigating optimal timing between surgery and RT start, but early experiences provide evidence of benefit for the use of EBRT (external beam radiation therapy) initially rather than at progression [80,90], and this approach is now accepted as the standard for MM. There is no study focused on the use of primary RT or SRS (stereotactic radiosurgery) in patients with grade 3 meningiomas. While SRS is a validated treatment for G1 meningioma, its role and efficacy in treating G3 are unclear. The administration of RT instead of surgery occurs as an obligate condition in patients where surgery is not an option because of the anatomical site of the tumor or when the performance status of the patient makes him unfit for surgery. For these reasons, this technique is often used in a bad prognosis population.

However, the effective benefit associated with this approach is uncertain [76,81,84,92]. Available evidence of primary RT is provided by retrospective series mainly focused on patients with grade 1 meningiomas of the specific anatomical site such as the optic nerve or skull base meningiomas. In these patients, primary RT (fractionated or SRS) is associated with a high percentage of local control (up to 95% at 5 years). However, data about the role of primary RT in more aggressive tumors are missing.

Patients with recurrent disease after prior radiation therapy in the same site have few therapeutic options. In these patients, surgery alone does not allow a significant local control improvement. Furthermore, the surgical bed is frequently too large to achieve an effective SRS. In these patients, the use of brachytherapy has been proposed. To date, the largest series reported consists of 42 patients with recurrent atypical or grade 3 meningioma [83]. In these patients, the introduction of I-125 permanent seed after re-resection was associated with a median time to progression of 11.4 months. However, there was significant toxicity associated with this treatment (19% of patients experiencing radiation necrosis; 14%, wound breakdown; and 7%, infections) [83].

Particle therapy with proton or carbon ion could reduce late toxicities in long-term survival, reducing the radiation delivered to the normal adjacent tissue. The increasing number of centers able to provide irradiation with heavy particles is leading to testing these techniques also in patients with meningiomas including anaplastic subtype. To date, the use of particle therapy, as well as proton radiation, is still experimental with several ongoing clinical trials (NCT01166321, NCT0269399, NCT01117844, NCT04278118).

## 8. Systemic Treatments

Although several agents have been tested in meningiomas refractory to surgery and radiation therapy, none of them suggested a clear clinical efficacy. Thus, to date, there is not a standard of care for systemic management of the disease, and the inclusion of these patients in clinical trials remains the best therapeutic option. The majority of clinical trials of systemic agents in meningiomas were small phase II trials without randomization (Table 2). Similarly, inclusion criteria allowed enrolment of refractory meningiomas regardless of their tumor grade. The study endpoint is also a debated issue even if the 6-month PFS and radiographic response are commonly adopted [50,102].

Temozolomide and irinotecan have been assessed in small phase II trials showing a modest efficacy [97,98]. More recently, trabectedin was investigated in a randomized phase II trial [110]. The comparator arm was the local standard of care and patients with recurrent grade 2 or 3 meningiomas. In this trial, there was no additional benefit in terms of OS and PFS with the administration of trabectedin. Of note, this study confirmed that the DNA methylation class of meningiomas was an independent prognostic factor for OS [110].

Meningiomas often express somatostatin receptors; therefore, somatostatin analogues and receptor radionuclide therapy have been tested in these tumors [96,108,113]. Trials investigating somatostatin analogues demonstrated a modest clinical activity of these compounds mainly resulting in reduced tumor growth. Octreotide shows to reduce cell proliferation but does not induce apoptosis of cancer cells [96,108,113]. In addition, peptide receptor radionuclide therapy failed to show a tumor shrinkage. Nonetheless, the use of ^177^Lu-DOTATOC was associated with a high percentage of stable disease [104].

In a meta-analysis carried out by Mirian C et al., 111 patients with treatment-refractory meningiomas received somatostatin receptor-targeted radiopeptide therapy [106]. Of the 19 patients with grade 3 meningioma included, the 6-month PFS was 0%, while the 1-year OS rate was 52% [106].

The evidence that meningioma is a largely vascularized tumor has led to the investigation of agents targeting angiogenesis. Inhibitors of the vascular endothelial growth factor (*VEGF*) such as bevacizumab [107,116] and the *VEGF* receptor (*VEGFR*) such as sunitinib and vatalanib [111] did not result in tumor responses but reached a high grade of 6 months PFS. In particular, sunitinib [103] administration in patients with refractory grade 2 or 3 meningiomas was associated with a 6-month PFS of 42% and a median OS of 24.6 months [103].

Other target molecules inhibiting the epidermic growth factor receptor (*EFGFR*) or the stem cell factor receptor (*KIT*) showed modest clinical efficacy in phase II mono-arm studies [109,114].

Meningiomas express the inactivation of the *NF2* in about 50% of cases. The inactivation of NF2 resulted in overexpression of the mammalian target of rapamycin complex 1 (*mTORC1*). The mTOR inhibitor everolimus has been tested in combination with bevacizumab and octreotide [99,112]. The CEVOREM trial assessed the combination between everolimus and octreotide. In this trial, the 1-year OS detected was 75%. Notably, about 80% of patients reported a decrease in the tumor growth rate of more than 50% [99].

Immune-checkpoint inhibitors (ICIs) are monoclonal antibodies able to restore an inhibited immune response against tumor cells. Two agents targeting the programmed death receptor 1 (*PD-1*) have been tested in refractory grade 2 or 3 meningiomas [91,94]. Pembrolizumab has been recently investigated in a small phase 2 trial on 25 patients [91]. This trial reaches its primary endpoint with a 6-month PFS of 48%. The *PD-1* inhibitor nivolumab failed to meet its primary endpoint and reach a 6-month PFS of 42.4% [94]; however, nivolumab administration was associated with a median OS of 30.9 months and led to a long-course radiographic response. Notably, both these studies reported that a subgroup of patients with refractory meningioma could be more likely to benefit from ICIs administration [91,94].

There are several novel molecules and treatments under investigation in refractory and grade 3 meningiomas. The only phase 3 trial assessing systemic agents which are currently recruiting patients is the POPLAR-NF2 trial (NCT05130866). This trial has a placebo as a comparator arm and is investigating the pan-histone deacetylase inhibitor REC 2282 in patients with germinal or sporadic NF2 mutated meningioma. The other two histone deacetylase inhibitors are under investigation in patients with refractory meningioma. These are Panobinostat (in combination with radiation therapy, NCT01324635) and AR-42 (NCT02282917). The *mTORC 1/2* dual inhibitor vistusertib showed promising clinical activity in preclinical [117] studies and is under investigation in two phase II clinical trials (NCT03071874, NCT02831257). The mitogen-activated protein kinase (*MEK*) inhibitor selumetinib (NCT03095248) and trametinib (NCT03631953) in combination with the PI3K inhibitor alpelisib and the cyclin-dependent kinase ribociclib (NCT02933736) are other agents under investigation. Tazemetostat is an (Enhancer Of Zeste 2 Polycomb Repressive Complex 2 Subunit) *EZH2* inhibitor which is currently under investigation in patients with *BAP-1* mutated meningioma (NCT02860286). Preclinical studies suggest that *NF2* mutated meningiomas could be more sensitive to the inhibition of the focal adhesion kinase (*FAK*) [118]; thus, a clinical trial involving a *FAK* inhibitor is currently ongoing (NCT02523014). Finally, other studies investigating ICIs are still ongoing and will respond to the clinical efficacy of PD-1 and CTLA-4 (cytotoxic T-lypmhocyte antigen 4) combination therapy (NCT02648997) as well as a combination of proton therapy and PD-1 inhibition with avelumab (NCT03267836). Notably, none of the mentioned trials is tailored for patients with grade 3 meningiomas, but about all of them allow the inclusion of patients with refractory meningiomas.

The Forkhead box M1 (*FOXM1*) transcription factor is an oncogenic driver often altered in high-grade meningioma and tumor aggressiveness [119]. Targets of FOXM1 have shown promising activity in patients with solid tumors, including gliomas [120,121]. It could be possible that these agents could be also assessed in MM patients in the coming future. NF2 mutated meningioma can often express the oncogene receptor *FGFR* (fibroblast growth factor receptor) and could be targeted by specific *FGFR* inhibitors [122]. To date, the FGFR inhibitor pemigatinib is under assessment in different primary central nervous system malignancies harboring activating *FGFR* alterations (NCT05267106).

## 9. Conclusions

Surgery and different RT approaches represent the milestone of grade 3 meningioma management. There is still a marginal role of systemic therapy. The emerging knowledge of the genomic alteration of the disease is an important achievement. This knowledge could lead to the development of effective drugs to modify the course of the disease.

## Figures and Tables

**Table 1:** Simpson grading for extent of meningioma resection.

Simpson Grade	Description
Grade 0	Complete tumor removal, plus removal of an additional 2–3 cm from the tumor insertion site
Grade I	Complete tumor removal, including any dural attachments or abnormal bone
Grade II	Complete tumor removal with coagulation of dural attachment
Grade III	Complete tumor removal without resection or coagulation of its dural attachment
Grade IV	Partial tumor removal
Grade V	Simple decompression with or without biopsy

**Table 2:** Prospective clinical trials investigating systemic treatments on meningioma. mOS: median overall survival, PFS-6mo: 6 months progression-free survival, OS-12mo: 12 months overall survival.

Experimental Arm	Phase and Number of Patients	Patients Enrolled and Population on Study	Outcome
Somatostatin Analogs			
Pasireotide [108]	Phase II, 34 patients	All grade recurrent meningiomas	Grade I: PFS-6mo 50%, mOS 26 months Grade II–III: PFS-6mo 17%, mOS 6.5 months
Octreotide [113]	Phase II, 9 patients	All grade recurrent meningiomas	PFS-6mo 44%, mOS 18.7 months
Long-acting octreotide [96]	Phase II, 16 patients	All grade recurrent meningiomas	PFS-6mo 44%, mOS 7.5 months
Octeotride + everolimus (CEVOREM trial) [99]	Phase II, 20 patients	All grade recurrent meningiomas refractory for surgery and radiotherapy	PFS-6mo 55%, OS-12mo 75%, Major decrease in growth rate of more than 50% in 78% of tumors
**Chemotherapy**			
Temozolomide [97]	Phase II, 16 patients	Grade I recurrent meningiomas	PFS-6mo 0%, mOS 7.5 months
Irinotecan [98]	Phase II, 16 patients	Grade I recurrent meningiomas	PFS-6mo 6%, mOS 7.0 months
Trabectedin (EORTC-1320-BTG) [110]	Randomized phase II trial (trabectedin versus local standard of care), 90 patients	Grade II–III meningiomas progressed after surgery and radiotherapy	No improvement of mPFS or mOS. PFS-6mo 21.1%, Median OS 11.37 months
Hyroxyurea + imatinib [105]	Phase II trial, 15 patients	All grade recurrent meningiomas	Prematurely closed due to slow accrual. No activity.
**Angiogenesis Inhibitors**			
Bevacizumab [100]	Phase II trial, 40 patients	All grade recurrent meningiomas	Grade I: PFS-6mo 87%, mOS 35.6 months Grade II: PFS-6mo 77%, mOS not reached Grade III: PFS-6mo 46%, mOS 12.4 months
Bevacizumab + everolimus [112]	Phase II trial, 17 patients	All grade recurrent meningiomas	PFS-6mo: 69%, mOS 23.8 months
Sunitinib [103]	Phase II trial, 38 patients	Grade II–III meningioma	PFS-6mo: 42%, mOS 24.6 months
Vatalanib [111]	Phase II trial, 22 patients	All grade recurrent meningiomas	PFS-6mo: 37.5%, mOS 23.0 months
**Target Agents**			
Erlotinib/Gefinitinib [109]	Phase II trial, 25 patients	All grade recurrent meningiomas	Grade I: PFS-6mo 25%, OS-12mo 50% Grade II–III: PFS-6mo 29%, OS-12mo 65%
Imatinib [114]	Phase II trial, 23 patients	All grade recurrent meningiomas	Grade I: PFS-6mo 45% Grade II–III: PFS-6mo 0%
**Immune-Checkpoint Inhibitors**			
Nivolumab [94]	Phase II, 25 patients	Grade II–III recurrent meningiomas	PFS-6mo 42.4%, mOS 30.9 months.
Pembrolizumab [91]	Phase II, 25 patients	Grade II–III recurrent meningiomas	PFS-6mo 48%
**Other Agents**			
Interferon alpha [95]	Phase II, 35 patients	Grade I recurrent meningiomas	PFS-6mo 54%, mOS 8 months
Mifepristone [101]	Phase III randomized (Mifepristone vs. placebo), 164 patients	All grade recurrent meningiomas	No difference with placebo in terms of overall survival and failure-free survival.

## Abbreviations

ADC:	apparent diffusion coefficient
AKT:	protein kinase B
BRCA:	BRCA-associated protein
CDKN2A/2B:	cyclin-dependent kinase inhibitor
CNS:	central nervous system
CR:	complete response
CSF:	cerebrospinal fluid
CT:	computed tomography
CTLA4:	cytotoxic T-lypmhocyte antigen 4
CTV:	clinical target volume
DWI:	diffusion-weighted imaging
EBRT:	external beam radiation therapy
EGFR:	epidermic growth factor receptors
EMA:	epithelial membrane antigen
EOR:	extent of resection
EORTC:	European Organization for Research and Treatment of Cancer
EZH2:	enhancer of zeste 2 polycomb repressive complex 2 subunit
FAK:	focal adhesion kinase
FGFR:	fibroblast growth factor receptor
FOXM1:	Forkhead box M1
GRADE:	grading of recommendations, assessment, development, and evaluation
GTR:	gross total resection
H3:	histone 3
ICIs:	immune-checkpoint inhibitors
KIT:	stem cell factor receptor
MEK:	mitogen-activated protein kinase
MM:	malignant meningioma
MR:	minor response
MRI:	magnetic resonance imaging
MTORC1:	mammalian target of rapamycin complex 1
NCDB:	National Cancer Database
NF2:	neurofibromatosis type 2 gene
OS:	overall survival
PD-1:	programmed death receptor 1
PD:	progressive disease
PFS:	progression-free survival
PIK3CA:	phosphatidylinositol-4,5-bisphosphate 3-kinase catalytic subunit alpha
PR:	partial response
RANO:	response assessment in neuro-oncology
rCBV:	relative cerebral blood volume
RT:	radiation therapy
RTOG:	radiation therapy oncology group
SD:	stable disease
SMO:	smoothened frizzled class receptor
SRS:	stereotactic radiosurgery
SSTR2A:	somatostatin receptor 2A
STR:	subtotal resection
TERT:	telomerase reverse transcriptase
TIL:	tumor-infiltrating lymphocytes
TRAF7:	tumor necrosis factor receptor associated factor 7
VEGF:	vascular endothelial growth factor
VEGFR:	vascular endothelial growth factor receptor
WHO:	World Health Organization
